# Report of a Case of Pressure Neuritis of Both Sciatic Nerves1Read before the Section on Medicine of the Buffalo Academy of Medicine, March 14, 1893.

**Published:** 1893-04

**Authors:** William C. Krauss

**Affiliations:** 382 Virginia Street; Buffalo, N. Y.


### REPORT OF A CASE OF PRESSURE NEURITIS OF BOTH SCIATIC NERVES.[1]

By WILLIAM C. KRAUSS, M. D., Buffalo, N. Y.

Name, S. B.; male; married; occupation, teamster; age, 35; height, five feet eight inches; weight, 175 pounds; complexion, dark; hair, brown; constitution, strong, healthy, well nourished.

*Antecedents.*—No history of any hereditary disease of any kind could be elicited.

*Early History.*—He was always a strong healthy boy; was never sick, nor given to complaining.

*Present History.*—In July, 1891, he noticed for the first time that his right testicle was swollen. It was neither painful nor sensitive, and was hard and firm to the touch. Continually increasing in size until it became as large as a goose egg, he decided to consult a surgeon. Up to this time he was able to carry on his work without any appreciable difficulty, but by nightfall was very weak and exhausted. His friends noticed that he was emaciating, his color was changing, and that he lacked much of the vim and buoyancy which characterized him in former days.

On September 27, 1891, the right testicle was removed and submitted to the writer for examination. The wound healed in a short time, and he again resumed his work. After four days' trial he was obliged to discontinue on account of the severe pain in the right hip extending down the dorsum of the leg into the foot. Not being benefited by the family physician, the writer was called in consultation December 20, 1891. The patient was found lying on his abdomen, his face flushed and bathed in perspiration, sobbing and groaning, apparently in much pain. On examination the right buttock was greatly swollen, reddened, painful and sensitive on pressure. In fact, pain on pressure existed throughout the whole course of the sciatic nerve. On account of the pain the patient would not flex the right leg, but held it fixed in an extended posi-

1. Read before the Section on Medicine of the Buffalo Academy of Medicine, March 14, 1893.

tion. There was also present anesthesia about the anus, inner side of the thigh and leg, and outer side of the right buttock. The left leg was unaffected; motility, sensation and the reflexes were to all intents normal.

There was, furthermore, retention of urine with pyuria, although catheterization was performed twice daily. The appetite was poor, bowels constipated, and he complained of an insatiable thirst. The pulse ranged from 110 to 130, the temperature 103° to 104° F. The inguinal glands were not swollen.

The patient's condition continued in this manner with but little variation until January 3, 1892, when the left buttock began to swell, and in a few days the left sciatic nerve also became painful and tender on pressure. There existed then, at this time, a double sciatica; swelling, redness, and tenderness over both buttocks; anesthesia extending over a considerable portion of the dorsum of both extremities; retention of urine with pyuria, a temperature of 103° to 104° F., and a pulse varying from 110 to 130. My suspicions, which were aroused on the first visit to the patient, namely, that there was a recurrent growth in the pelvis, now seemed to be materialized, and proof was further adduced by finding a hard, dense nodular mass dorsad of the rectum. The patient slowly continued to sink; the buttocks were growing larger, until they seemed ready to burst; defecation became painful and irregular; and in the first week of March, 1892, there developed a pulmonary edema, death occurring on the fourteenth. The last rectal examination was made a few days prior to his death, and the whole pelvis was found filled with a hard, firm, unyielding mass. An autopsy was not permitted, and the diagnosis of the neoplasm must be made by inference. The enlarged scrotum, removed September 27, 1891, proved to be a fibro-sarcoma with predominance of small spindle cells, and hence we may assume that the mass in the pelvis was an osteo-sarcoma of the sacrum. The clinical history of the case bears out this diagnosis. Whether the sacral tumor was the result of metastases from the scrotal tumor, or *vice versa*, cannot, of course, be determined. The pulmonary affection may also have been the result of metastases, with edema as a complication.

Double or bilateral sciaticas are, as a rule, very rare, and when they do occur, do not depend, in the great majority of cases, upon any of the etiological factors which we are prone to associate with unilateral sciaticas. These latter are generally either of rheumatic, neuralgic, malarial, toxic, or traumatic origin, while the former, in nearly all instances, are produced by pressure upon the lumbar enlargement of the nerve trunks by some neoplasm, hemorrhage, or inflammatory process in the sacrum or pelvis; consequently, the prognosis and treatment of the one differs materially from that of the other.

In the case just reported, the symptoms kept pace with the growth of the tumor. The right lateral mass of the sacrum was in all probability the primary seat of the growth in the pelvis. As it enlarged, it encroached upon the course of the right sacral plexus —especially the greater and small sciatic and pudic nerves,—then extending to the left side of the sacrum, it affected in like manner the nerve trunks of the left sacral plexus. No attempt was made to remove the mass from the pelvis. The treatment of the case was palliative throughout, the pain being controlled satisfactorily with galvanism and opium.

382 Virginia Street.

